# Stereotactic Evacuation Performed During the Subacute Phase Improves Functional Independence Measurement Scores in Patients With Putaminal Hemorrhage

**DOI:** 10.7759/cureus.83341

**Published:** 2025-05-02

**Authors:** Tomoko Eto, Yusuke Otsu, Yoh Yamakawa, Shin Yamashita, Terukazu Kuramoto, Etsuro Kawaguchi, Yu Hasegawa, Kiyohiko Sakata, Motohiro Morioka

**Affiliations:** 1 Neurosurgery, Omuta City Hospital, Omuta, JPN; 2 Neurosurgery, Kurume University Hospital, Kurume, JPN; 3 Neurosurgery, Kurume University, Kurume, JPN; 4 Central Division of Radiology, Omuta City Hospital, Omuta, JPN; 5 Pharmaceutical Sciences, International University of Health and Welfare, Okawa, JPN

**Keywords:** cognitive impairment, functional independence measurement, putaminal hemorrhage, stereotactic evacuation, subacute phase

## Abstract

Introduction: Surgery for putaminal hemorrhage during the acute phase does not improve motor and cognitive impairments. We investigated the effect of stereotactic evacuation performed seven days after the onset of putaminal hemorrhage on the Functional Independence Measurement (FIM) score, compared with that of conservative treatment.

Methods: We included 203 patients admitted for putaminal hemorrhage treatment between January 2012 and December 2022. Of these, 28 patients with putaminal hemorrhage (computed tomography classification IVa: putaminal hemorrhage into the anterior and posterior limbs of the internal capsule without ventricular perforation) who did not undergo surgery in the acute phase were divided into stereotactic evacuation (n = 14) and conservative treatment (n = 14) groups. We then evaluated the total, motor, and cognitive FIM scores at three months post admission.

Results: Greater average hematoma volume (p = 0.06) and greater perihematomal edema volume (p = 0.07) on admission were observed in the stereotactic evacuation group. We performed stereotactic evacuation 13.6 ± 3.4 days after admission, with an average hematoma removal rate of 91.4%. The total and cognitive FIM scores at three months improved in the stereotactic evacuation group.

Conclusion: Our findings indicate that stereotactic evacuation in the subacute phase has better functional outcomes, especially in patients with cognitive impairment. Minimally invasive surgery may be a promising option to improve patients’ prognoses.

## Introduction

Patients with putaminal hemorrhage, the most frequent subtype of intracerebral hemorrhage, show significant motor and cognitive impairments in their functional prognosis, and determining which treatment is beneficial for them is challenging. Hemorrhage with moderate neurological deficits and a volume greater than 31 cm^3^ or brain herniation can be considered an indication for surgical treatment with craniotomy in the acute period [1-3]. Patients who do not undergo surgical treatment in the acute period may experience prolonged impaired consciousness and worsening of neurological findings in the subacute and chronic periods due to secondary brain injuries, mostly due to hematoma degradation product-related cytotoxicity and perihematomal edema [4-6]. However, the surgical indications and optimal timing for reducing brain injury remain undetermined.

Early surgical treatments for hemorrhage, such as craniotomy and minimally invasive surgery, have not been shown to significantly improve functional outcomes [7,8]. In our institution, we have performed stereotactic evacuation of hematomas for patients with putaminal hemorrhage during the subacute phase. We believe that the clot is softer and more easily evacuated in the subacute phase, with an associated reduction in the rebleeding rate. Furthermore, prolonged perihematomal edema, which is involved in the pathophysiology of the attendant brain injury, may be controlled by the evacuation of the hematoma.

This study aimed to examine the beneficial effects of minimally invasive surgery performed during the subacute phase on putaminal hemorrhage. Therefore, we compared the patients who underwent surgery in the subacute period with those who underwent conservative treatment and evaluated their prognosis using the Functional Independence Measurement (FIM) score.

## Materials and methods

Patient selection and characteristics

The study protocol was approved by the ethics committee of our institution (Omuta City Hospital; Approval No. 2322), and the study was conducted at a single institution. A total of 203 patients with putaminal hemorrhage were admitted to our hospital between January 2012 and December 2022. Among them, 29 patients underwent craniotomy for hematoma removal within 24 hours after admission, and 36 patients underwent stereotactic evacuation during the subacute period. The remaining 138 patients were treated conservatively.

Stereotactic evacuation during the subacute period (seven to 18 days after onset) was performed for patients whose consciousness disturbance was prolonged (Glasgow Coma Scale < 13) or whose neurological deficits did not improve within seven to 14 days of admission, based on the decisions of the attending physicians. This treatment strategy has been consistently applied at our institution.

**Figure 1:**
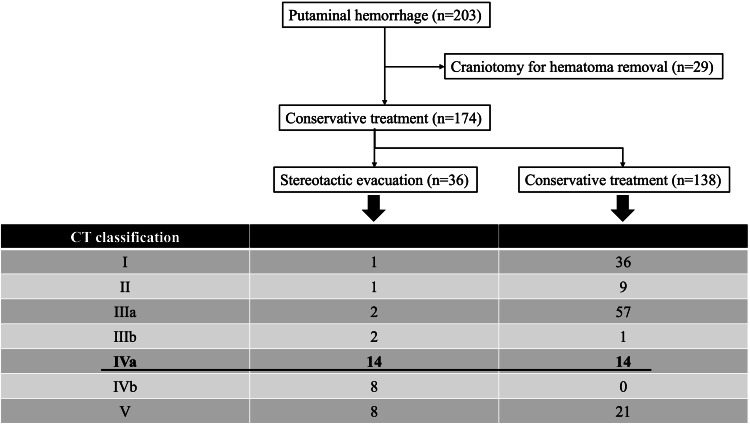
Flowchart of the study. A total of 203 patients with putaminal hemorrhage were admitted to our hospital. Twenty-nine patients underwent craniotomy for hematoma removal within 24 hours after admission. Thirty-six patients underwent stereotactic evacuation in the subacute phase.

For the purpose of the present study, we retrospectively selected patients with hemorrhages extending into both the anterior and posterior limbs of the internal capsule without ventricular perforation (CT classification grade IVa; n = 28) [9], and divided them into stereotactic evacuation (n = 14) and conservative treatment (n = 14) groups according to the above-mentioned treatment policy (Figure 1). We then evaluated the prognosis of the two groups three months after admission using the FIM score.

Data collected from their medical records included age, sex, preoperative use of antithrombotic drugs, CT image findings (pre- and postoperative hematoma volume, edema volume, and laterality), pre- and postoperative modified Rankin Scale (mRS) and FIM scores, and the postoperative course.

Measurement of hematoma size and perihematomal edema volume

All patients underwent a plain CT upon admission. A follow-up CT was performed on the day after the surgery in the stereotactic evacuation group, while patients in the conservative treatment group underwent a second CT approximately two weeks after admission. The CT images were transferred to a workstation (Synapse Vincent, Fujifilm Corp., Tokyo, Japan) for post-processing. Intracranial axial slices (5.0 mm thick) were traced semi-manually by an experienced neurosurgeon, and the perihematomal edema volume was measured by assigning Hounsfield units ranging from 60 to 80 to hematoma and those ranging from 5 to 33 to edema [10].

Stereotactic evacuation

We performed a stereotactic evacuation using Komai’s stereotactic frame (Mizuho Ika Kogyo, Tokyo, Japan). We chose the frontal or parietal approach depending on the size of the hematoma and the direction of its development, and took single-track aspiration. Aspiration was undertaken using a 3 mm needle in the external diameter based on the preoperative CT scan, with a target hematoma removal rate of at least 80%. No drainage was used during the procedure.

FIM

The FIM score was evaluated at admission and three months after admission. It consists of 18 daily activity items, including 13 motor items and five cognitive items, with a total score that ranges from 18 to 126. Each item is rated on a seven-point scale ranging from 1 (completely dependent) to 7 (independent) based on the level of independence. A score of 91 points on the motor FIM and 35 points on the cognitive FIM represents optimal performance [11]. 

Statistical analysis

Data were expressed as mean ± standard deviation. A non-paired t-test or Mann-Whitney U test was used to compare data between the two groups (age, sex, hematoma volume, perihematomal edema volume, mRS scores, Glasgow Outcome Scale (GOS), and FIM scores), while other variables were analyzed using Fisher’s exact test. The John Macintosh Project (JMP) software version 16 (SAS Institute Inc., Cary, NC, USA) was used for statistical analyses. Statistical significance was set at p < 0.05.

## Results

Baseline clinical characteristics

As shown in Table 1, there were no differences in the mRS scores, anti-thrombotic drug use before disease onset, Glasgow Coma Scale (GCS) on admission, total and cognitive FIM score on admission, laterality of the hemorrhage, level of consciousness at six days after admission, or number of patients with deteriorated level of consciousness between the groups. However, the stereotactic evacuation group had significantly more females (p = 0.02) and a younger mean age (p = 0.02) than the conservative treatment group. Similarly, the stereotactic evaluation group tended to have better motor FIM scores (p = 0.08), a larger average hematoma (p = 0.06), and a larger perihematomal edema volume on admission (p = 0.07) than the conservative treatment group.

**Table 1:** Comparison of patients’ characteristics between the stereotactic evacuation and conservative treatment groups of patients with CT classification IVa putaminal hemorrhage. Values are expressed as mean ± standard deviation. Statistical comparisons were performed using the unpaired t-test, Mann–Whitney U test, or Fisher’s exact test. Statistical significance was defined as p < 0.05. Pre: pre-admission; mRS: modified Rankin Scale; FIM: Functional Independence Measurement; GCS: Glasgow Coma Scale.

Characteristics	Conservative treatment	Stereotactic evacuation	p-value
No. of cases	14	14	
Age (years)	66.4 ± 11.7	64.9 ± 14.4	0.02
Sex ratio (male: female)	11:3	4:10	0.02
Pre- anti-thrombotic drugs, n (%)	1 (7.1%)	2 (14.3%)	>0.99
mRS before onset	1.1 ± 1.4	0.6 ± 1.5	0.10
GCS on admission			>0.99
13 ≦ GCS ≦ 15	10	9	
GCS < 13	4	5	
FIM on admission			
Total	23.6 ± 14.5	27.3 ± 14.9	0.15
Motor	14.9 ± 7.2	15.7 ± 6.7	0.08
Cognitive	8.6 ± 7.9	11.6 ± 9.0	0.22
CT finding			
Laterality (left: right)	7:7	8:6	>0.99
Average hematoma volume on admission (cm^3^)	23.7 ± 11.5	31.7 ± 10.1	0.06
Average perihematomal edema volume on admission (cm^3^)	46.5 ± 14.0	60.1 ± 23.4	0.07
GCS 6 days after admission			0.44
13 ≦ GCS ≦ 15	10	7	
GCS < 13	4	7	
Deterioration of the level of consciousness			0.24
Yes	3	7	
No	11	7	

Hematoma removal rate within the subacute phase and the acute phase

**Figure 2:**
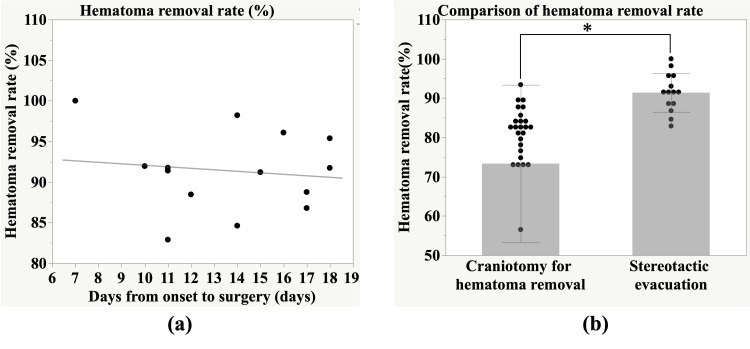
Hematoma removal rate within the subacute phase and the acute phase. (a) No significant correlation was observed between the number of days from onset to surgery and the hematoma removal rate (r = -0.12, p = 0.66). (b) The average hematoma removal rate in the subacute phase (91.4%) was significantly higher than that in the acute phase (73.3%) (p < 0.01). Statistical comparison was performed using the unpaired t-test. Statistical significance was defined as p < 0.05.

Figure 2A shows the rate of change in hematoma volume after stereotactic evacuation. We performed stereotactic evacuation 13.6 ± 3.4 days after admission, with an average hematoma removal rate of 91.4%. The average operative time was 1.45 hours, the average volume of operative bleeding loss was 12 ml, and no cases of rebleeding were observed. A follow-up CT scan was performed the day after surgery; on average, it was performed 14.6 ± 3.4 and 13.2 ± 4.8 days after admission in the stereotactic evacuation and conservative treatment groups, respectively (p = 0.37). There was no significant association between the number of days from disease onset to surgery and the hematoma removal rate (r = -0.12, p = 0.66).

Figure 2B compares the hematoma removal rate with craniotomy hematoma removal in the acute phase and stereotactic evacuation in the subacute phase. The average hematoma removal rate by craniotomy hematoma removal was 73.3%. The hematoma removal rate was significantly higher for stereotactic evacuation in the subacute phase (p < 0.01).

Prognosis

**Figure 3:**
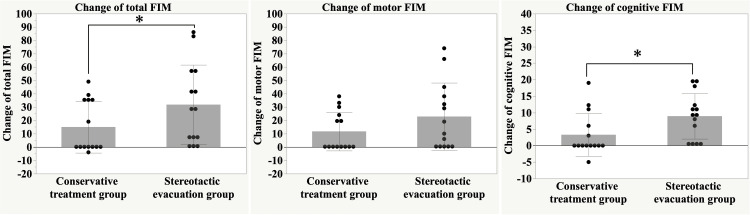
Change in Functional Independence Measurement (FIM) scores. The stereotactic evacuation group showed significantly greater improvement in total and cognitive FIM scores at three months after admission compared to the conservative treatment group. p < 0.05, determined by the Mann-Whitney U test. Statistical significance was defined as p < 0.05.

As shown in Table 2, the postoperative perihematomal edema volume and the changes observed between the first CT, GOS at discharge, mRS, and motor FIM, and those at three months postoperatively were not significantly different between the two groups. However, the total and cognitive FIM scores at three months and the changes in the two items were significantly better in the stereotactic evacuation group than in the conservative treatment group (Figure 3). Total FIM scores at three months were 58.1 ± 32.9 and 39.2 ± 27.1 in the stereotactic evacuation and conservative treatment groups (p = 0.048). Cognitive FIM scores at three months were 38.6 ± 24.4 and 26.6 ± 18.5 in the stereotactic evacuation and conservative treatment groups (p = 0.02). When comparing pre-treatment and three-month post-treatment values, the stereotactic evacuation group showed a 112.8% increase in total FIM (from 27.3 to 58.1), a 145.9% increase in motor FIM (from 15.7 to 38.6), and a 76.7% increase in cognitive FIM (from 11.6 to 20.5). In contrast, the conservative treatment group showed a 66.1% increase in total FIM (from 23.6 to 39.2), a 78.5% increase in motor FIM (from 14.9 to 26.6), and a 38.4% increase in cognitive FIM (from 8.6 to 11.9).

**Table 2:** Comparison of patient prognosis between the stereotactic evacuation and conservative treatment groups of patients with CT classification IVa putaminal hemorrhage. Values are expressed as mean ± standard deviation. Statistical comparisons were performed using the unpaired t-test or the Mann-Whitney U test. Statistical significance was defined as p < 0.05. GOS: Glasgow Outcome Scale; mRS: modified Rankin Scale; FIM: Functional Independence Measurement.

Characteristics	Conservative treatment	Stereotactic evacuation	p-value
Postoperative perihematomal edema volume (mm^3^)	77.1 ± 23.3	85.7 ± 37.7	0.48
Changes in perihematomal edema volume (%)	73.5 ± 63.1	43.0 ± 42.6	0.11
GOS at discharge	3.0 ± 0.4	3.1 ± 0.5	0.50
mRS at 3 months	4.3 ± 0.8	3.8 ± 0.9	0.11
FIM at 3 months			
Total	39.2 ± 27.1	58.1 ± 32.9	0.048
Motor	26.6 ± 18.5	38.6 ± 24.4	0.10
Cognitive	11.9 ± 9.7	20.5 ± 9.5	0.02
Changes in FIM			
Total	15.0 ± 19.4	31.8 ± 29.8	0.04
Motor	11.7 ± 14.4	22.9 ± 25.2	0.19
Cognitive	3.3 ± 6.5	8.9 ± 6.9	0.02

Representative case presentation

**Figure 4:**
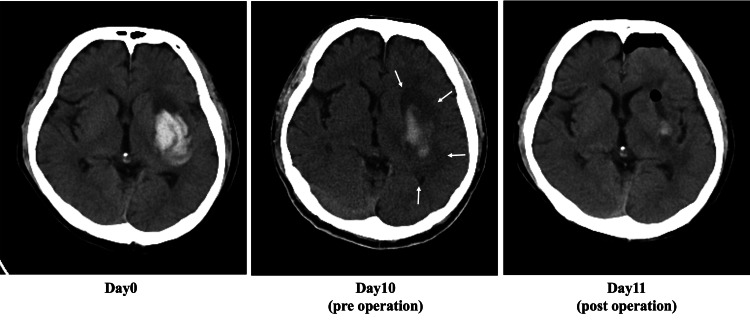
A representative case of left putaminal hemorrhage. The patient underwent stereotactic evacuation on day 10. The hematoma and perihematomal edema volumes were reduced by 91.9% and 77.9%, respectively. There was no postoperative rebleed.

The representative case shown in Figure 4 is that of a 42-year-old male with a left putaminal hemorrhage. On admission, the hematoma volume was 23.1 cm^3^, and the perihematomal edema volume was 34.5 cm^3^. The patient’s consciousness gradually deteriorated after admission. We performed stereotactic hematoma evacuation because the perihematomal edema had expanded on follow-up CT on day 10. On the day after surgery, the patient’s level of consciousness improved. A postoperative CT scan showed that 91.9% of the hematoma was removed, and the perihematomal edema volume was reduced by 77.9% compared to the CT obtained at admission. The patient was then transferred to a rehabilitation hospital. The total FIM score was 25 (motor FIM: 13; cognitive FIM: 12) on admission, and it increased to 66 (motor FIM: 42; cognitive FIM: 24) three months later.

## Discussion

This study found that stereotactic evacuation performed within the subacute period was effective for treating patients with class IVa putaminal hemorrhage. This is evident from the following results of the comparison between the stereotactic evacuation and the conservative group: (1) we achieved subtotal removal of the hematoma, and the total FIM scores in the surgery group significantly improved; and (2) improvement in cognitive FIM scores rather than motor function was observed in the stereotactic evacuation group. However, there were no differences in the level of consciousness between the two groups at six days after admission.

Although surgical treatment for patients with intracerebral hemorrhage is available, early evacuation craniotomy does not result in functional improvement [8]. In addition, minimally invasive surgery such as stereotactic evacuation and endoscopic removal, which have the advantages of inhibiting traumatic injury to the normal brain, may be an alternative surgical option [12]. No significant effect of early intervention was observed in a previous study, even if the hematoma size was reduced by 69% [7]. This suggests that primary injuries due to intracerebral hemorrhage should not be treated with acute surgical management [9].

In this study, patients with putaminal hemorrhage underwent stereotactic evacuation more than seven days after disease onset. To the best of our knowledge, no previous study has evaluated the usefulness of treatment in the subacute phase of this disease. Since the goal and timing of surgery differ between the acute and subacute phases, the current results should not be interpreted as a direct comparison with acute-phase interventions. During the acute period (within three days after bleeding), the hematoma clot was hard, and more than 80% of the hematoma was removed in only 43.8% of cases in a previous study [13]. In contrast, our current results demonstrated that 91.3% of the hematoma was removed without rebleeding. Although endoscopic hematoma removal offers the advantage of hemostasis through coagulation, spontaneous hemostasis has already been achieved in the subacute phase. Stereotactic hematoma removal can be performed safely and with minimal invasiveness because of shorter operative time and lower intraoperative bleeding [14]. Hematomas in the subacute period (approximately seven days after disease onset) are softened and can be easily aspirated, as is the case with subacute subdural hematomas; thus, the hematoma removal rate should be high. It may be better to perform stereotactic evacuation after seven days from the onset of the disease to prevent secondary brain injury, as there was no correlation between the number of days from onset to surgery and the hematoma removal rate of stereotactic evacuation in our patients. The optimal timing for stereotactic evacuation is as early as possible in the subacute phase, around seven days after onset, to prevent secondary brain injury and facilitate early rehabilitation. In the pathophysiology of chronic brain injury due to intracerebral hemorrhage, an important factor is secondary brain injury, which is mainly caused by blood clots and perihematomal edema formation [15]. Because there was no change in perihematomal edema volume between the groups, the reduction in the pathological deterioration caused by clots, including inflammatory responses and oxidative stress, may benefit our patients. Taken together, we suggest that stereotactic evacuation performed for patients with class IVa putaminal hemorrhage during the subacute period is safe and achieves a higher hematoma removal rate than surgery performed during the acute period. This results in a better prognosis.

Putaminal hemorrhage can induce motor paralysis, sensory disturbances, and cognitive dysfunction, depending on the location and size of the hematoma and perihematomal edema [16]. In this study, stereotactic evacuation was the most commonly performed in putaminal hemorrhage class Ⅳa and was included in the study. Eight patients also underwent stereotactic evacuation in grade IVb (hemorrhages extending into the anterior and posterior limbs of the internal capsule without ventricular perforation) and grade V (hemorrhages extending into the thalamus and hypothalamus), but there was no improvement in functional prognosis. Previous studies have reported that hematoma volume and age are important risk factors for cognitive impairment in patients with putaminal hemorrhage [16]. Based on our findings, the hematoma volume and perihematomal edema were marginally larger in the stereotactic evacuation group than in the conservative treatment group, although there were significantly younger patients in the former group. A previous study showed that fiber anisotropy at the fornix and the apparent diffusion coefficient of the neural tracts were correlated with cognitive function in patients with putaminal hemorrhage [17]. In our study, the postsurgical perihematomal edema volume was similar between the groups; however, an obvious reduction in the hematoma volume was observed after stereotactic evacuation. Although the patients only underwent CT scans and we could not demonstrate detailed structural changes and differences in the neural tracts, the reduction in the hematoma size after minimally invasive surgery performed during the subacute phase may contribute to structural changes in the brain areas that control cognitive function, such as the fornix. In contrast, the motor FIM scores in the stereotactic evacuation group did not improve after hematoma reduction due to the irreversible injury caused by the putaminal hemorrhage. In this regard, further studies are needed to clarify this relationship using magnetic resonance imaging.

This study has several limitations. Firstly, there is possibly patient selection bias. Our surgical indications were as follows: prolonged altered consciousness and/or no improvement in neurological findings within seven to 14 days of admission. However, the final decision was made by the attending physician, considering the patient’s background and other factors. In addition, the mean age and sex distribution differed between the two groups, with younger and more female patients included in the stereotactic evacuation group. The reason for this imbalance remains unclear, but it may reflect the attending physicians’ preferences or other unmeasured factors and may have influenced the outcomes. Secondly, there were cases in which the FIM score differed between the initial and three-month follow-up. Patients are usually transferred to rehabilitation hospitals after completing the acute to chronic period of treatment, and in some cases, evaluation after three months is implemented at the rehabilitation hospital. Finally, the number of cases was small. No studies have evaluated the effectiveness of stereotactic hematoma evacuation performed during the subacute phase of putaminal hemorrhage. A larger randomized clinical trial should be conducted to determine the effectiveness of stereotactic hematoma evacuation performed during the chronic phase of putaminal hemorrhage.

## Conclusions

Our findings suggest that stereotactic evacuation performed during the subacute phase for patients with CT classification IVa putaminal hemorrhage contributes to significantly better functional outcomes, particularly in terms of cognitive improvement. In this study, we compared subacute stereotactic evacuation with conservative treatment, not with acute phase hematoma removal. Compared with conservative treatment, subacute stereotactic evacuation was associated with higher hematoma removal rates, minimal invasiveness, and a lower risk of complications, including rebleeding. The cognitive benefit observed may reflect the reduction of secondary brain injury caused by prolonged clot presence, including inflammation and oxidative stress.

Given the favorable outcomes demonstrated in this study, stereotactic evacuation during the subacute phase may be considered a promising treatment strategy for patients who are not eligible for acute surgical intervention. Future studies with larger sample sizes and advanced imaging techniques are needed to further elucidate the optimal timing and indications for this approach and to confirm its efficacy in broader patient populations.
